# Cost-effectiveness analysis of sintilimab plus bevacizumab biosimilar compared with lenvatinib as the first-line treatment of unresectable or metastatic hepatocellular carcinoma

**DOI:** 10.1186/s12913-022-08661-4

**Published:** 2022-11-17

**Authors:** Ting Zhou, Xintian Wang, Yingdan Cao, Lan Yang, Zijing Wang, Aixia Ma, Hongchao Li

**Affiliations:** School of International Pharmaceutical Business, China Pharmaceutical University, Nanjing, China

**Keywords:** Hepatocellular carcinoma, Sintilimab, Lenvatinib, Cost-effectiveness, PD-1

## Abstract

**Background:**

In recent years, programmed cell death protein-1 inhibitors, including sintilimab, have significantly prolonged the overall survival time of patients with unresectable or metastatic hepatocellular carcinoma (HCC); however, the cost-effectiveness of sintilimab is unclear. The aim of this study was to assess the cost-effectiveness of sintilimab plus bevacizumab biosimilar compared with lenvatinib as first-line treatment in patients with unresectable or metastatic HCC.

**Methods:**

A lifetime partitioned survival model was developed to conduct a cost-effectiveness analysis of sintilimab plus bevacizumab biosimilar vs. lenvatinib for advanced HCC from a Chinese healthcare system perspective. The clinical and safety data were derived from two recent randomized clinical trials, the ORIENT-32 and REFLECT studies. Utility data were obtained from previous studies. Long-term direct medical costs and quality-adjusted life-years (QALYs) were predicted. Deterministic and probabilistic sensitivity analyses were performed to verify the robustness of the model.

**Results:**

Compared with lenvatinib, combination therapy with sintilimab and bevacizumab biosimilar yielded an additional 0.493 QALYs at a higher cost ($33,102 vs. $21,037) (2021 US dollars). This resulted in a deterministic incremental cost-effectiveness ratio (ICER) of $24,462 per QALY in the base-case analysis. The ICERs were sensitive to the utility of post-progression and the cost of bevacizumab biosimilar. A lower ICER was estimated when the dose of bevacizumab biosimilar decreased from 15 mg to 7.5 mg per kilogram in the scenario analysis. In the probabilistic sensitivity analysis, the probability of being cost-effective for sintilimab treatment at willingness-to-pay (WTP) thresholds of one ($12,516) and three times the gross domestic product per capita in China ($37,547) were 11.6% and 88.6%, respectively.

**Conclusion:**

Sintilimab plus bevacizumab biosimilar is likely to be a cost-effective treatment option as a first-line treatment for unresectable or metastatic HCC in China when WTP threshold is over $23,650.

**Supplementary information:**

The online version contains supplementary material available at 10.1186/s12913-022-08661-4.

## Introduction

Liver cancer is a malignancy that occurs in liver cells or intrahepatic bile duct cells. Worldwide, liver cancer is the sixth most common cancer by incidence and the third most fatal cause of tumors[1]. The estimated rates of new liver cancer cases and deaths in China were 26.92 per 100,000 and 13.72 per 100,000 in 2015[2]. Existing studies show that the incidence of liver cancer in China is currently much higher than the world average level, although it may show a downward trend in the future[3]. Hepatocellular carcinoma (HCC) is the most common type of primary liver cancer, accounting for 85-90% of cases[4]. Approximately 40% of HCC patients have unresectable or metastatic HCC at the time of diagnosis and are ineligible for radical surgical resection and insensitive to chemotherapy.

Recently, the approval of molecularly targeted drugs and immune checkpoint inhibitors has provided new treatment options for patients with unresectable or metastatic HCC. Targeted drugs, such as lenvatinib, are recommended as first-line treatment regimens by the Guidelines of the Chinese Society of Clinical Oncology Hepatocellular Carcinoma 2020[4]. In addition, sintilimab (a programmed cell death protein-1 inhibitor) plus bevacizumab or biosimilar (a vascular endothelial growth factor inhibitor) combination therapy has been upgraded as a first-line treatment (grade II) recommendation for advanced HCC in the Guidelines of the Chinese Society of Clinical Oncology Immune Checkpoint Inhibitor Clinical Practice 2021[5].

Sintilimab, a programmed cell death protein-1 inhibitor, activates the human immune system to generate a tumor immune response[6]. In June 2021, the combination treatment of sintilimab with bevacizumab biosimilar was approved for the treatment of first-line unresectable or metastatic HCC in China by the National Medical Products Administration (NMPA), mainly based on the ORIENT-32 study[6]. To date, it has been the first and only programmed cell death protein-1 inhibitor approved for this indication in China. The ORIENT-32 study shows that the efficacy of sintilimab plus bevacizumab biosimilar is significantly better than that of sorafenib[6]. There have been several studies compared cost-effectiveness between sintilimab plus bevacizumab biosimilar therapy and sorafenib in advanced HCC in China [7, 8]. The results show that sintilimab combination therapy is a cost-effective option for the first-line treatment of unresectable or metastatic HCC in China [7, 8].

Similarly, the oral multiple receptor tyrosine kinase inhibitor lenvatinib was approved for advanced HCC by the NMPA in 2018. The phase III clinical trial REFLECT showed that lenvatinib is non-inferior to sorafenib in terms of improving patient overall survival (OS) [9]. Although sintilimab plus bevacizumab biosimilar therapy and lenvatinib have shown good efficacy in clinical trials and are recommended by Chinese guideline[5], a comparison of the cost- and clinical-effectiveness of these regimens has not yet been undertaken in a Chinese setting. Therefore, this study aimed to evaluate the cost-effectiveness of sintilimab plus bevacizumab biosimilar vs. lenvatinib for the first-line treatment of unresectable or metastatic HCC in China.

## Methods

A cost-effectiveness analysis was conducted by developing a three-state partitioned survival model (PSM) from the Chinese healthcare system perspective over a lifetime horizon. Quality-adjusted life-years (QALYs) were used as an indicator to measure the health outcomes of patients. Patients’ treatment costs and health outcomes were estimated to calculate the incremental cost-effectiveness ratios (ICERs). Costs were adjusted to 2021 US dollars (1 US dollar = 6.47 RMB[10]). Future costs and health outcomes were discounted at 5% annually (0.28% per three weeks) according to the Chinese pharmacoeconomic evaluation guidelines[11]. One ($12,516) and three times the gross domestic product (GDP) per capita of China in 2021 ($37,547) were used as the willingness-to-pay (WTP) thresholds[11]. This analysis followed the Consolidated Health Economic Evaluation Reporting Standards reporting guidelines[12].

### Model overview

PSMs are widely applied in the economic evaluation of cancer. This model mainly uses the proportion of individuals in different health states derived from survival curves and avoids the calculation of independent transition probabilities[13]. The PSM is usually recommended when individual patient data (IPD) are available[14].

**Fig. 1:**
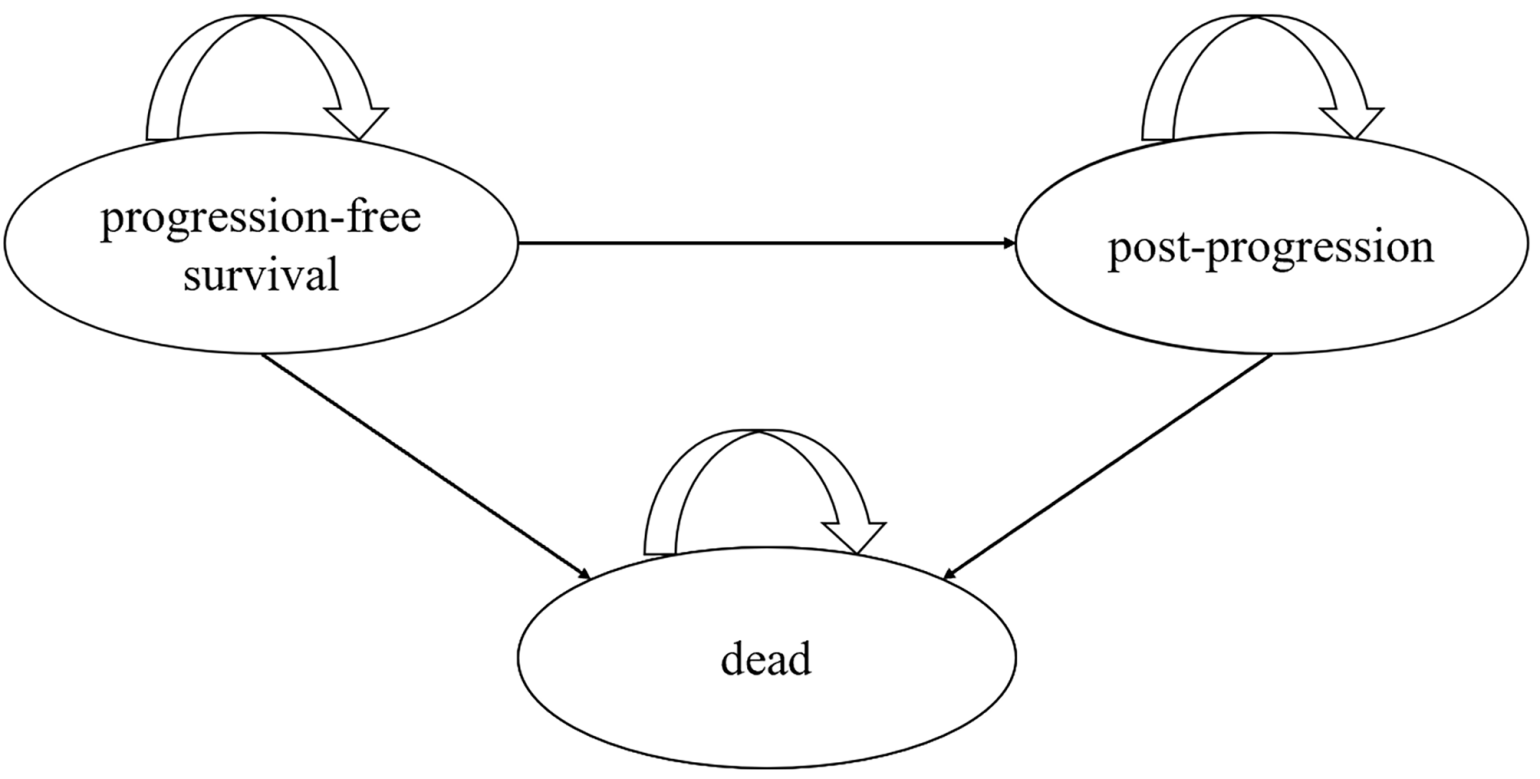
The structure of partitioned survival model

In this study, a PSM was developed in Microsoft Excel 2019 based on the IPD of the ORIENT-32 study, the published results of the REFLECT study, previous literature and clinical expert opinion. The model included three mutually exclusive health states: progression-free survival (PFS), post-progression (PP) and death (Fig. 1). It was assumed that all patient population cohorts entered the model in the PFS state. Patients in PFS state could remain in the PFS state or transitioned to the PP state or died after receiving treatment. Patients in the PP state could stay in the same state or move to the death state. The length of the translation cycle for the model was set to 3 weeks, which was consistent with the treatment cycle duration for sintilimab strategy.

### Patient cohort

The baseline characteristics of the patients modelled in the PSM were assumed to be in line with the ORIENT-32 and REFLECT studies[6, 9]. For patients in the PFS state, the intervention arm received sintilimab intravenously (200 mg) and bevacizumab biosimilar intravenously (15 mg/kg) on the first day of each cycle (every 3 weeks). For the control group, patients received oral lenvatinib 12 mg/day. After disease progression, patients in both groups received subsequent treatment until death.

### Model input

#### Clinical efficacy data

The clinical efficacy data used in PSMs were mainly obtained from the ORIENT-32 study and the REFLECT study[6, 9].

The randomized, open-label phase III study ORIENT-32[6] enrolled 571 Chinese patients with unresectable or metastatic HCC who had not received systemic treatment in the past. The patients were randomly assigned at a ratio of 2:1 to receive sorafenib or sintilimab plus bevacizumab biosimilar. In the ORIENT-32 study, sintilimab plus bevacizumab biosimilar showed a significantly longer median PFS (4.6 months vs. 2.8 months, hazard ratio (HR) = 0.56, p < 0.0001) and an overall survival benefit (median not reached vs. 10.4 months) than sorafenib.

The REFLECT study[9] was a global multicenter, randomized, open-ended, noninferiority phase III clinical trial conducted in 20 countries. A total of 954 patients with unresectable HCC were included, of which nearly 300 patients were from China. The patients were randomly assigned to receive lenvatinib or sorafenib for treatment. The median survival time of lenvatinib was noninferior to that of sorafenib (13.6 months vs. 12.3 months, HR = 0.92), and treatment with lenvatinib resulted in a significant improvement in PFS compared with treatment with sorafenib (7.4 months vs. 3.7 months, HR = 0.66, p < 0.0001). The results suggested that lenvatinib showed better efficacy in the first-line treatment of unresectable HCC than sorafenib. The baseline characteristics of ORIENT-32 and REFLECT study were presented in Supplementary Table 1.

Due to the lack of a head-to-head trial comparing sintilimab plus bevacizumab biosimilar and lenvatinib, we used an indirect comparison method to calculate the HRs of PFS and OS for the two regimens. For the best available data, sorafenib was used as a control arm to perform an indirect comparison based on the ORIENT-32 study[6] and the REFLECT study[9]. A Bayesian network meta-analysis (NMA) was conducted using sorafenib as the connecting therapy. The HRs of OS and PFS between sintilimab arm and lenvatinib were calculated in NMA. The HR of PFS for the sintilimab arm vs. the lenvatinib arm was 0.856 (95% confidence interval [CI] 0.798–0.910), and the HR of OS was 0.618 (95% CI 0.546–0.708), which were obtained by indirect comparison.

Using the IPD of the sintilimab arm, Kaplan-Meier curves of PFS and OS were drawn. Six standard parameter distributions, including exponential, gamma, Weibull, Gompertz, log-normal and log-logistic distributions, were used to fit the Kaplan-Meier curves. The best fitting distribution was selected according to the Akaike information criterion, Bayesian information criterion and visual inspection[15, 16]. The smaller the AIC and BIC values are, the better the fit of the distribution. The testing results of the different distributions are shown in Supplementary Tables 2 and 3. Log-normal was selected as the best fitting distribution for the PFS curve and OS curve of the sintilimab arm according to AIC and BIC. Based on HR adjustment, the lenvatinib arm followed the same distribution as the sintilimab arm. The reconstruction of Kaplan-Meier curves and Bayesian NMA were performed using R 3.6.1 software.

In addition, the incidence of adverse events (AEs) was used as an indicator to measure the safety of the treatment regimens. AEs related to drug treatment with an incidence of ≥ 5% and a grade ≥ 3 in the ORIENT-32 study and the REFLECT study were included in the model in terms of management costs and effect on utility decremental (Table 1). As a result, hypertension and platelet count reduction were considered in sintilimab combination treatment, while hypertension, decreased platelet count, proteinuria, increased blood bilirubin and elevated aspartate aminotransferase were considered in the lenvatinib arm in our model. All efficacy and AE probabilities were converted to three-week cycle probabilities.

**Table 1:** Model Inputs

Parameters	Base value		Distribution	Range	Source
**Drug costs ($)**					
Sintilimab (100 mg)	166.92		Gamma	133.54-166.92	[20]
Bevacizumab biosimilar (100 mg)	177.13		Gamma	123.99-177.13	[20]
Lenvatinib (4 mg)	16.69		Gamma	11.68–16.69	[20]
**Subsequent treatment ($)**					
Sintilimab + Bevacizumab	442.29		Gamma	353.83-530.74	[6]
Lenvatinib	631.81		Gamma	505.45-758.17	[6]
**End-of-life care ($)**	2132.67		Gamma	888.10-6108.44	[21]
**Cost of follow-up per visit ($)**					
Computer tomography	90.43		Gamma	64.91-146.83	Medical service price database of 11 provinces in China
Blood biochemistry	24.27		Gamma	13.76–38.02	
Blood routine	1.58		Gamma	0.77–2.94	
Urine routine	0.67		Gamma	0.15–1.39	
Myocardial enzyme test	5.13		Gamma	3.09–7.73	
alpha feprotein test	3.18		Gamma	2.16–4.64	
**Cost of drug administration ($)**					
Diagnosis	5.13		Gamma	3.09–7.73	Medical service price database of 11 provinces in China
Intravenous injection	1.73		Gamma	0.46–4.64	
Nursing	1.27		Gamma	0.77–1.85	
Bed	2.05		Gamma	1.64–2.47	
**Cost of AEs per event ($)**					
Hypertension	3.42		Gamma	2.73–4.10	Expert opinion
Proteinuria	22.10		Gamma	17.68–26.52	Expert opinion
Platelet count decreased	176.55		Gamma	141.24-211.86	Expert opinion
Elevated bilirubin	77.28		Gamma	61.82–92.74	Expert opinion
Elevated aspartate aminotransferase	77.28		Gamma	61.82–92.74	Expert opinion
**HR (Sintilimab + Bevacizumab versus Lenvatinib)**					
PFS	0.856		Log-normal	0.798–0.910	[6, 9]
OS	0.618		Log-normal	0.546–0.708	[6, 9]
Probability of AEs (per cycle)*					
Sintilimab + Bevacizumab					
Hypertension	0.72%		Beta	0.58-0.87%	[6]
Platelet count decreased	0.33%		Beta	0.27-0.40%	[6]
Lenvatinib					
Hypertension	1.32%		Beta	1.06-1.58%	[9]
Proteinuria	0.35%		Beta	0.28-0.41%	[9]
Platelet count decreased	0.29%		Beta	0.23-0.35%	[9]
Elevated bilirubin	0.40%		Beta	0.32-0.48%	[9]
Elevated aspartate aminotransferase	0.29%		Beta	0.23-0.35%	[9]
**Health state utility**					
PFS state	0.745		Beta	0.730–0.760	[17]
PP state	0.678		Beta	0.655–0.701	[17]
**Disutility of AEs**					
Hypertension	-0.12		Beta	-0.10 to -0.14	[18]
Proteinuria	-0.12		Beta	-0.10 to -0.14	[18]
Decreased platelet count	0.00		Beta	0.00	[19]
Increased blood bilirubin	0.00		Beta	0.00	[19]
Elevated aspartate aminotransferase	0.00		Beta	0.00	[19]
**Discount rate (per cycle)**	0.28%		Beta	0.00-0.44%	

PFS: progression-free survival; PP: post-progression; AE: adverse event, OS: overall survival; HR: hazard ratio

*Assumed the AEs occurred in first year

#### Health utility

The health state utility values were obtained from the *National Institute for Health and Care Excellence technology appraisal guidance TA551-2018*[17]. The utility values of the PFS state and PP state were 0.745 and 0.678, respectively. The utility was measured by EQ-5D-3L instrument and collected from global multi-center HCC patients received first line treatment of lenvatinib or sorafenib in REFLECT clinical trial. In addition, the disutility associated with the incidence of AEs in the ORIENT-32 study and REFLECT study described above was obtained from published literature (Table 1)[18, 19].

#### Cost data

This analysis was conducted from a Chinese healthcare system perspective, and only direct medical costs were included. In the PSM, costs of treatment, AE management, subsequent treatment, drug administration, follow-up and end-of-life care were estimated according to each health state (Table 1).

The treatment costs for sintilimab, bevacizumab biosimilar and lenvatinib were only considered in the PFS state, and these prices were obtained from the MENET database[6, 20]. Meanwhile, we assumed that patients were treated with sintilimab plus bevacizumab biosimilar for up to two years even if the disease had not progressed for a small proportion of patients according to clinical expert consultation.

After the disease progressed, patients received subsequent treatment. The costs of subsequent treatment were calculated based on the IPD of the ORIENT-32 study[20]. Due to the lack of relevant data on subsequent treatments after lenvatinib, the cost was assumed to be the average of the subsequent treatment cost of the intervention group and the control group in the ORIENT-32 study. The drug administration cost and follow-up cost in the PFS and PP states were obtained from the medical and health service price databases of 11 representative provinces (low-, middle-, and high-income levels) in China and verified by clinical experts. The cost of end-of-life care was identified from the literature[21].

### Scenario analysis

In real-world clinical practice, a lower dose of bevacizumab and biosimilar are commonly used in China due to the intolerance of treatment. The dose is often reduced to 7.5 mg/kg from the standard of 15 mg/kg. Therefore, a scenario analysis was performed to explore the cost-effectiveness of sintilimab plus bevacizumab biosimilar after the decrease in bevacizumab biosimilar dose to 7.5 mg/kg.

### Sensitivity analysis

To explore the uncertainty of the model, one-way sensitivity analysis and probabilistic sensitivity analysis (PSA) were conducted.

The one-way sensitivity analysis was carried out by varying each parameter at one time within the 95% confidence interval and assuming a variance of ± 20% for the baseline value when such data were unavailable. The analysis result was presented as a tornado diagram.

The PSA was used to explore the effect of simultaneous changes in all variables on outcomes. We assumed that the probability and utility value followed the beta distribution, HR followed log-normal distribution, and that the cost data followed the gamma distribution. For PSA, 5,000 Monte Carlo iterations were performed and a cost-effectiveness plane and cost-effectiveness acceptability curve (CEAC) were produced.

## Results

### Base-case analysis

For Chinese unresectable or metastatic HCC patients, compared with lenvatinib, sintilimab plus bevacizumab biosimilar yielded an additional 0.493 QALYs (1.431 QALYs vs. 0.938 QALYs), corresponding to an incremental cost of $12,065 ($33,102 vs. $21,037). The ICER was $24,462 per QALY gained, and the value was lower than the WTP of two times the GDP per capita in China (Table 2).

**Table 2:** Results of the Base-case and Scenario Analyses

		COST ($)	QALY	LYs	ICER($/QALY)
**Base case**					
Sintilimab + Bevacizumab		33,102	1.431	2.04	24,462
Lenvatinib		21,037	0.938	1.32	-
**Scenario**					
Sintilimab + Bevacizumab		24,752	1.431	2.04	7,533
Lenvatinib		21,037	0.938	1.32	-

QALY: quality-adjusted life-year; LYs: life-years; ICER: incremental cost-effectiveness ratio

### Scenario analysis

In the scenario analysis to address real-world clinical practice and reduce the dose of bevacizumab biosimilar from 15 mg/kg to 7.5 mg/kg, sintilimab was associated with an incremental benefit of 0.493 QALYs (1.431 QALYs vs. 0.938 QALYs) and an incremental cost of $3,715 ($24,752 vs. $21,307) compared with lenvatinib over a lifetime horizon when clinical efficacy remained the same across bevacizumab biosimilar doses. The ICER was $7,533 per QALY gained.

### Sensitivity analysis

**Fig. 2:**
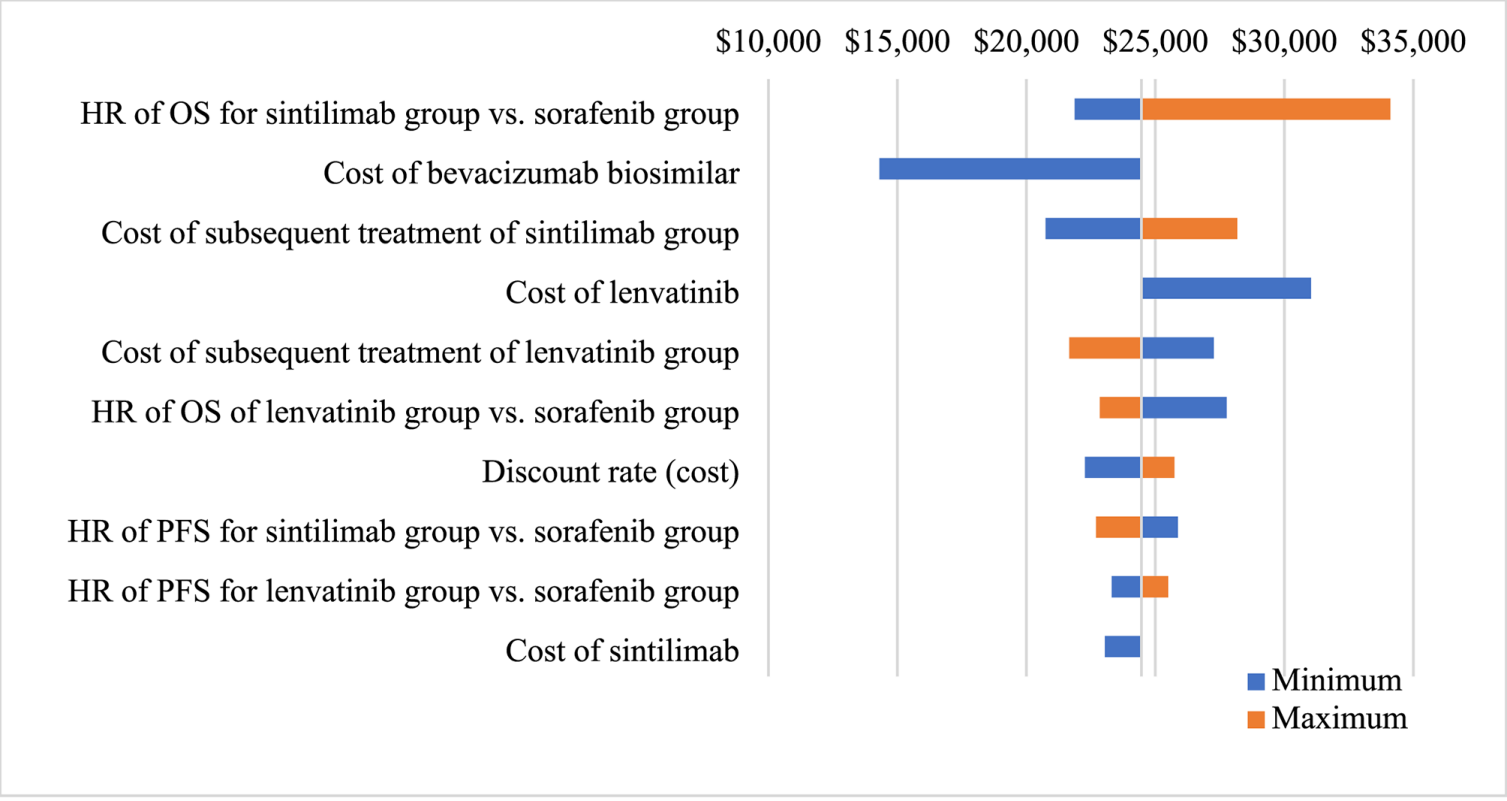
Tornado diagram of the one-way sensitivity analyses. PFS: progression-free survival; OS: overall survival; HR: hazard ratio

The results of the one-way sensitivity analysis are shown in the tornado diagram in Fig. 2. The ICER was mainly sensitive to HR of OS for sintilimab arm vs. sorafenib arm, the cost of bevacizumab biosimilar and the cost of subsequent treatment in the sintilimab group. Within the parameter range, the ICER result varied from $14,304/QALY in the best case to $34,113/QALY in the worst case.

**Fig. 3:**
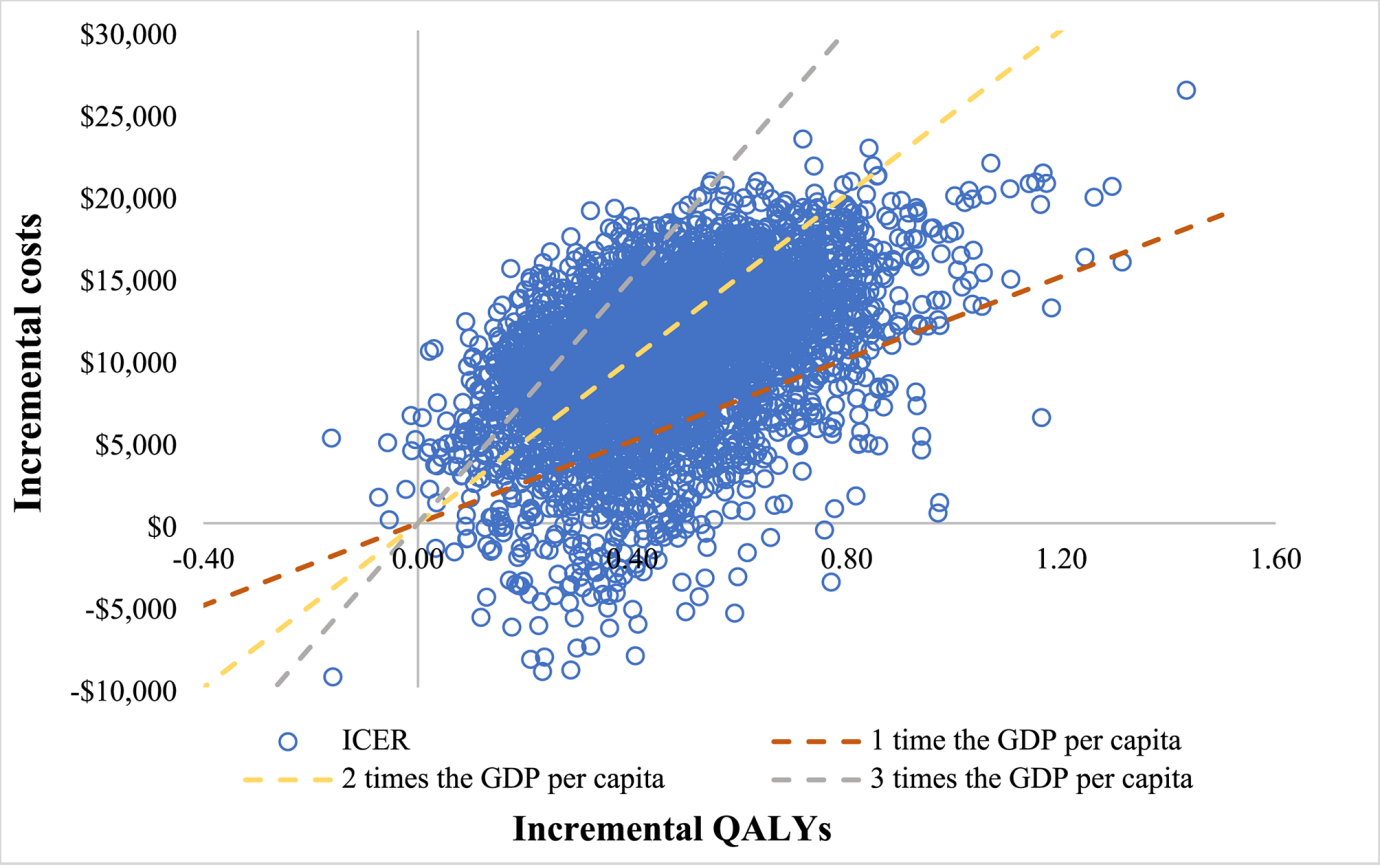
Cost-effectiveness plane. QALY: quality-adjusted life-year; ICER: incremental cost-effectiveness ratio; GDP: gross domestic product

**Fig. 4:**
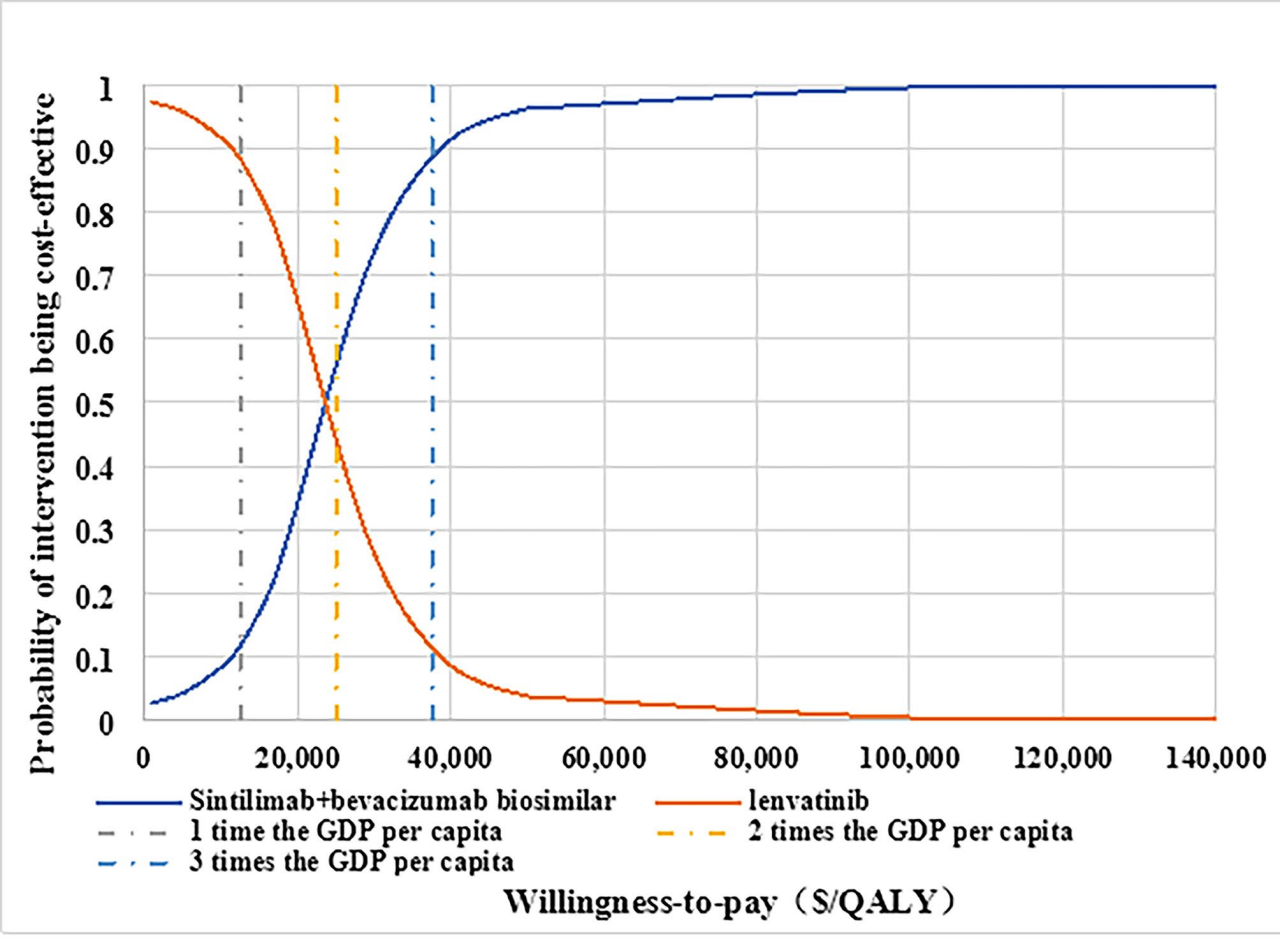
Cost-effectiveness acceptability curve. QALY: quality-adjusted life-year; GDP:gross domestic product

In PSA, the cost-effectiveness plane and the cost-effectiveness acceptability curve for the results of 5,000 Monte Carlo iterations are presented in Figs. 3 and 4. Most ICERs fell in the first quadrant of the cost-effectiveness plane and were below the trend line representing a WTP threshold of three times the GDP per capita in 2021 in China and over half of the ICERs below the trend line at two times the GDP per capita in China, which indicated that sintilimab had a higher probability of being cost-effective at these WTP thresholds. The CEAC illustrated that the probability of sintilimab strategy being more cost-effective than lenvatinib was 11.6%, 55.8%, 88.6% under the WTP thresholds of $12,516/QALY (GDP per capita in 2021), $25,031/QALY (two times the GDP per capita in 2021) and $37,547/QALY (three times the GDP per capita in 2021), respectively. The sintilimab strategy had a higher probability being cost-effective than lenvatinib when WTP is over $23,650 in China.

## Discussion

In this study, a PSM was constructed to evaluate the cost-effectiveness of sintilimab plus bevacizumab biosimilar compared with lenvatinib for the first-line treatment of unresectable or metastatic HCC in China from a Chinese healthcare system perspective.

The main findings of our analysis indicated that sintilimab plus bevacizumab biosimilar could improve the patients’ quality of life but incurred higher cost compared with lenvatinib in China. According to the cost and QALY breakdown, an increased survival time in the PFS state led to higher QALYs for patients who received sintilimab and bevacizumab biosimilar, although the treatment cost was also higher. In addition, the longer OS observed in PSM for the sintilimab arm than the lenvatinib arm was mainly contributed by patients living in the PP state. As a result, patients who received sintilimab combination therapy and progressed achieved almost twice as many QALYs as patients in the lenvatinib group in the PP state. The greater effect of sintilimab resulted in a longer duration of therapy; therefore, a higher average cost is inevitable.

The cost of bevacizumab biosimilar accounts for a higher proportion (50%) of the total cost of the sintilimab group than others, which may have a great impact on the cost-effectiveness results. In the scenario analysis, the cost-effectiveness of sintilimab plus bevacizumab biosimilar was significantly improved when the dose of bevacizumab biosimilar was reduced to be in line with real-world clinical practice. However, same efficacy assumption with base-case analysis might overestimate the results. With the approval of more biosimilars of bevacizumab by the NMPA, the price of bevacizumab biosimilar may decrease in the near future and thus improve the probability of being cost-effective in combination with sintilimab. The one-way sensitivity analysis results also illustrated that the ICER was sensitive to the cost of bevacizumab biosimilar.

There have been several published economic evaluations of lenvatinib in different settings; however, they mainly compared lenvatinib with sorafenib. These analyses showed that across the world, including Japan[22, 23], Australia[19], Canada[18, 24], and China[25], lenvatinib is more cost-effective than sorafenib for HCC patients. Wen et al[26] and Su et al[27] focused on the cost-effectiveness of an ICI plus a VEGF inhibitor vs. a tyrosine kinase inhibitor (atezolizumab plus bevacizumab vs. sorafenib) for the treatment of advanced HCC in China. Similar to our study, the results of these studies showed that compared with sorafenib, atezolizumab and bevacizumab combination therapy generated additional QALYs but incurred higher costs, resulting in ICERs of $145,546/QALY[26] and $169,223/QALY[27], respectively. Atezolizumab plus bevacizumab is unlikely to be a cost-effective option in China. However, the sintilimab plus bevacizumab biosimilar strategy could reduce the ICER and improve the probability of being cost-effective because of the lower cost of sintilimab.

HCC imposes heavy disease and economic burdens on patients and society in China. Although significant progress has been made with the approval of new treatments in China, the prognosis of HCC is still unsatisfactory. To accelerate access to new pharmaceutical interventions for patients and reduce the financial burden, the Chinese National Healthcare Security Administration launched price negotiations for “high value but high price” drugs in 2017. To be listed in the National Drug Reimbursement Listing, the pharmaceutical manufacturer needs to reach a price agreement with the Chinese National Healthcare Security Administration. In 2021, sintilimab was covered by national medical insurance in the National Drug Reimbursement Listing for HCC and had a 62% reduction in the retail price.

This study has several limitations. (1) An indirect comparison was required due to the lack of a head-to-head clinical trial, and the IPD of the REFLECT study was unavailable. Given the heterogeneity, the results of this study may be biased. (2) Without local utility data of local HCC patients, the best available utility data are mainly based on the multi-country HCC population reported in the NICE TA551. (3) The OS curves of the patients enrolled in the ORIENT-32 study are not mature; thus, the PFS and OS curves were extrapolated using parameter distribution fitting, which may increase the uncertainty of the results. (4) This study was performed from a health system perspective, and only the direct medical cost of the patient was considered. Model validation is needed in the future.

## Conclusion

This study suggests that compared with lenvatinib, sintilimab plus bevacizumab biosimilar combination therapy is a cost-effective option in the first-line treatment of unresectable or metastatic HCC in China from the perspective of the Chinese health system when WTP threshold is over $23,650.

## Electronic supplementary material

Below is the link to the electronic supplementary material.

Supplementary Material 1

## Data Availability

All data analyzed during this work were included in this article and supplementary materials.

## **Abbreviations**

HCC: hepatocellular carcinoma
QALYs: quality-adjusted life-years
PSA: probabilistic sensitivity analysis
ICER: incremental cost-effectiveness ratio
OS: overall survival
PSM: partitioned survival model
GDP: gross domestic product
WTP: willingness-to-pay
IPD: individual patient data
PFS: progression-free survival
PP: post-progression
HR: hazard ratio
AEs: adverse events
CEAC: cost-effectiveness acceptability curve
