# Supplementary material for: Cost-effectiveness analysis of sintilimab plus bevacizumab biosimilar compared with lenvatinib as the first-line treatment of unresectable or metastatic hepatocellular carcinoma
Source: BMC Health Serv Res. 2022 Nov 17;22:1367. doi: 10.1186/s12913-022-08661-4 (PMC9673291; doi:10.1186/s12913-022-08661-4)
Supplement: Supplementary file 1 — Supplementary Material 1 [file 12913_2022_8661_MOESM1_ESM.docx]

**Supplementary Material.**

Table 1. Patient baseline characteristics in ORIENT-32 study and REFLECT study.

| **Characteristic** | **sintilimab+bevacizumab biosimilar group (n=380)** | **lenvatinib group(n=478)** |
| --- | --- | --- |
| Median Age (range) | 53 (21-82) | 63 (20-88) |
| Male sex | 334 (88%) | 405 (85%) |
| ECOG performance status |  |  |
| 0 | 183 (48%) | 304 (64%) |
| 1 | 197 (52%) | 174 (36%) |
| Macrovascular invasion, extrahepatic metastasis, or both |  |  |
| Yes | 303 (80%) | 329 (69%) |
| No | 77 (20%) | 149 (31%) |
| BCLC stage |  |  |
| B | 56 (15%) | 104 (22%) |
| C | 324 (85%) | 374 (78%) |
| Race | Chinese |  |
| White | 0 (0%) | 135 (28%) |
| Asian | 380 (100%) | 334 (70%) |
| Other | 0 (0%) | 9 (2%) |
| Hepatitis B virus infection | 359 (94%) | 251 (53%) |
| Hepatitis C virus infection | 6 (2%) | 91 (19%) |

ECOG Eastern Cooperative Oncology Group, BCLC Barcelona Clinic Liver Cancer

| Table 2. Akaike information criterion and Bayesian information criterion for parametric models of sintilimab+bevacizumab biosimilar PFS | | |
| --- | --- | --- |
| sintilimab+bevacizumab PFS | AIC | BIC |
| Exponential | 1492.02 | 1495.96 |
| Gamma | 1482.17 | 1490.05 |
| Geompertz | 1493.61 | 1501.49 |
| Weibull | 1488.16 | 1496.04 |
| Log-Logistic | 1459.22 | 1467.11 |
| **Log-Normal** | **1439.93** | **1447.81** |
| AIC Akaike information criterion, BIC Bayesian information criterion, PFS progression-free survival. | | |

| Table 3. Akaike information criterion and Bayesian information criterion for parametric models of sintilimab+bevacizumab biosimilar OS | | |
| --- | --- | --- |
| sintilimab+bevacizumab OS | AIC | BIC |
| Exponential | 1052.56 | 1056.50 |
| Gamma | 1020.10 | 1027.98 |
| Geompertz | 1038.51 | 1046.39 |
| Weibull | 1023.42 | 1031.30 |
| Log-Logistic | 1018.99 | 1026.87 |
| **Log-Normal** | **1015.59** | **1023.47** |

AIC Akaike information criterion, BIC Bayesian information criterion, OS overall survival.
